# Control of actin polymerization *via* reactive oxygen species generation using light or radiation

**DOI:** 10.3389/fcell.2022.1014008

**Published:** 2022-09-23

**Authors:** Tetsuya Ishimoto, Hisashi Mori

**Affiliations:** ^1^ Department of Molecular Neuroscience, Faculty of Medicine, University of Toyama, Toyama, Japan; ^2^ Research Center for Idling Brain Science, University of Toyama, Toyama, Japan; ^3^ Research Center for Pre-Disease Science, University of Toyama, Toyama, Japan

**Keywords:** actin, reactive oxygen species, laser, ultraviolet, polymerization, chromophore-assisted light inactivation

## Abstract

Actin is one of the most prevalent proteins in cells, and its amino acid sequence is remarkably conserved from protozoa to humans. The polymerization-depolymerization cycle of actin immediately below the plasma membrane regulates cell function, motility, and morphology. It is known that actin and other actin-binding proteins are targets for reactive oxygen species (ROS), indicating that ROS affects cells through actin reorganization. Several researchers have attempted to control actin polymerization from outside the cell to mimic or inhibit actin reorganization. To modify the polymerization state of actin, ultraviolet, visible, and near-infrared light, ionizing radiation, and chromophore-assisted light inactivation have all been reported to induce ROS. Additionally, a combination of the fluorescent protein KillerRed and the luminescent protein luciferase can generate ROS on actin fibers and promote actin polymerization. These techniques are very useful tools for analyzing the relationship between ROS and cell function, movement, and morphology, and are also expected to be used in therapeutics. In this mini review, we offer an overview of the advancements in this field, with a particular focus on how to control intracellular actin polymerization using such optical approaches, and discuss future challenges.

## Introduction

Actin is a 42 kDa cytoskeletal protein with a highly conserved sequence from lower organisms to humans. Actin filaments form a double helix and are thinner and relatively shorter than microtubules and intermediate filaments. Polymerized actin is prevalent at the cell’s periphery, where it interacts with actin-binding proteins and assumes several shapes, including filopodia, and lamellipodia. Compared to microtubules and filaments of intermediate diameter, polymerization-depolymerization occurs more frequently and is consequently the cytoskeleton that plays the most significant role in cell motility.

Actin polymerization and depolymerization are known to be altered by the oxidation of actin or actin-binding proteins (Wilson et al., 2016; Varland et al., 2019; Balta et al., 2020). Consequently, numerous data indicate that actin polymerization can be induced by adding hydrogen peroxide (Boardman et al., 2004; Lee et al., 2004; Kim et al., 2009; Cinq-Frais et al., 2015). However, it is challenging to spatially and temporally regulate the polymerization and depolymerization of actin in tissues or within cells. However, optical approaches, such as laser irradiation, can regulate the irradiated area with more precision and allow for more sophisticated control. Accordingly, optical approaches will be required in the future, for the optical manipulation of actin in therapeutic applications.

**FIGURE 1:**
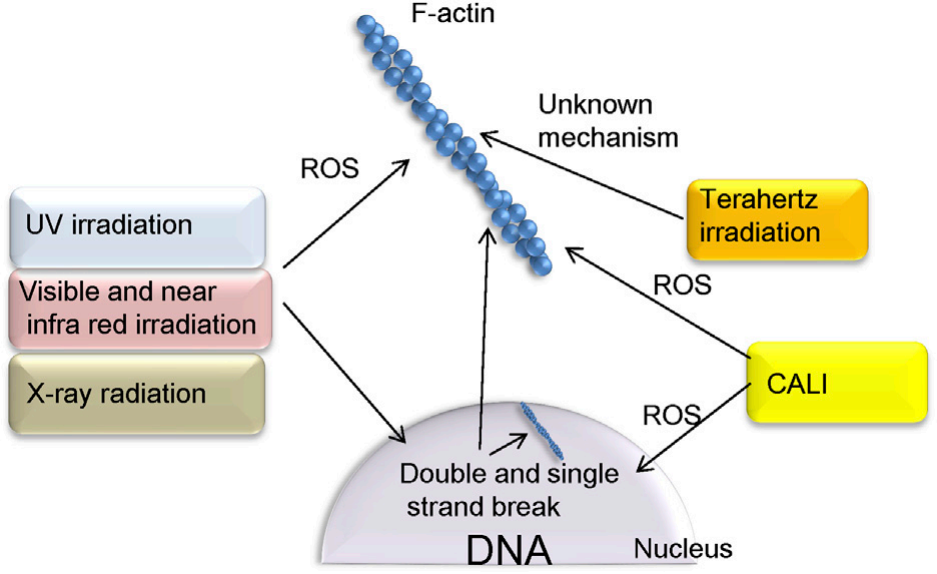
Methods of controlling actin polymerization via ROS, as presented in this paper. UV, NIR, and radiation, depending on their parameters and cell type, also cause double strand breaks in DNA as well as ROS production. Terahertz irradiation is considered noninvasive and affects actin polymerization via unknown mechanism. In the CALI method, proteins that fuse with KillerRed can be localized to specific sites in the cell. This allows ROS to act on specific targets such as F-actin and nuclei.

In this mini review, we have summarized methods of optically regulating actin polymerization-depolymerization (Figure 1). Many of these are believed to be influenced by the generation of ROS by an optical stimulus, which oxidizes actin or actin-binding proteins. Also presented are instances in which the generation of ROS has not always been shown to be the cause of actin control.

### Ultraviolet irradiation

Ultraviolet (UV) light is a component of sunlight, and the majority of UV rays that reach the ground are UVA (320–400 nm) and UVB (290–320 nm). UV light induces erythema and tanning as acute effects, and skin cancer and photoaging as chronic consequences, in human skin (Krutmann, 2001; Bachelor and Bowden, 2004). In addition to direct impacts on DNA, the production of ROS is the primary source of these effects. UVB is claimed to generate ROS by activating NADPH oxidase and the respiratory chain reaction (Masaki et al., 1995; Jurkiewicz and Buettner, 1996). On the other hand, UVA is reported to induce the activation of NADPH oxidase 1 (Valencia and Kochevar, 2008) and the photosensitization of the advanced glycation end product (Masaki et al., 1999). Kovacs et al. observed that UVB irradiation (310 nm, 0.59 mW/cm^2^, 50 mJ/cm^2^) of keratinocytes in culture induced ROS production by fluorescent observation using fluorescent probe 20,70-dichlorofluorescein diacetate and Western blotting and that actin polymerization decreased. According to their findings, the generated ROS induces actin depolymerization. In addition, they discovered that the keratinocyte growth factor inhibits ROS production (Kovacs et al., 2009). In B16 melanoma cells, exposure to narrow-band UVB (100 mJ/cm^2^) for 30 min activated Rac1 and elevated stress fibers. This paper did not claim that actin polymerization was produced by ROS, however, this is considered a possibility since UVB creates ROS (Wang et al., 2013). After 2 h of UVA (365 nm, 18.7 J/cm^2^, 381 μW/cm^2^, 60 min) exposure, dysplastic keratinocytes generate more filopodia than normal cells. This irradiation simultaneously produced focal contact stronger than normal cells. These findings imply that dysplastic keratinocytes are more sensitive to UVA than regular keratinocytes (Niculite et al., 2018). Analysis of F-actin by rhodamine-phalloidin in the lens epithelium of smooth dogfish subjected to (365 nm, 2.5 mW/cm^2^) UV for 18 h revealed the destruction of basal actin filaments. Simultaneously, the proportion of G-actin increased. Thus, the authors concluded that the lens contains UV-vulnerable actin filaments (Zigman et al., 1992).

It is known that UV has genotoxicity. In nature, UV-C, which does not fall to the ground, has the strongest genotoxicity, but UV-B, which reaches the ground, also has genotoxicity because it is absorbed by DNA. UV-A may also have genotoxicity indirectly due to ROS produced by irradiation (Douki, 2020).

### Visible and near-infrared irradiation (NIR)

Red and near-infrared light have been employed in everyday life as very familiar low-level illumination. These lights are utilized in therapies known as low-level laser or photobiomodulation therapies, which are utilized primarily in the dermatological and orthopedic disciplines (Farivar et al., 2014; Glass, 2021). These therapies utilize light itself, as opposed to the heat produced by the light. There are numerous hypotheses on the mechanism of action, however, some propose that it involves the production of ROS. Accordingly, there are numerous reports of ROS production at low levels of light (Alexandratou et al., 2002; Lavi et al., 2003; Chen et al., 2011; George et al., 2018). Kao et al. found that blue light (473 nm, 10 min, and 160 μW) induced neurite retraction in neuroblastoma N2a cells, whereas red light (650 nm, 60 min, and 25 μW) stimulated neurite regeneration with actin propagation. They also concluded that blue light-induced contraction is caused by ROS production (Kao et al., 2019). Irradiation of bone marrow cells with a long-wavelength laser (808 nm, 60 J/cm^2^, and 60 s) resulted in the thickening of actin filaments, which ran parallel to form an expanded membrane. However, the authors did not specify if this was associated with ROS development (Amaroli et al., 2021). de Magalhaes et al. found that the number of actin filaments decreased in mouse 3T3 cells, 5 min after irradiation (300 s) with 625 nm (115 mW/cm^2^, 35 J/cm^2^), or 808 nm (125 mW/cm^2^, 38 J/cm^2^) light. This rapid response indicates a correlation with ROS production. Furthermore, this reduction is recovered 24 h after irradiation (de Magalhaes et al., 2020).

By mixing photosensitizers and lasers, attempts have been made to modify actin fibers in cells. Photosensitizers are molecules that release ROS when exposed to light, allowing for the efficient production of ROS solely at the laser-irradiated location (Tada and Baptista, 2015). MCF7 breast cancer cells were exposed to a 650 nm laser (20 mW) in conjunction with the photosensitizer 5-5-(4-N, N-diacetoxylphenyl-10, 15, 20-tetraphenylporphyrin) (DTPP). Actin fibers increased 3 h after irradiation and returned to normal 12 h later. In contrast, there was no change in the expression level of actin protein. Thus, the authors speculated that singlet oxygen is produced *via* DTPP, which affects actin fiber (Wang et al., 2014). Wu et al. (2016) utilized sinoporphyrin sodium as a photosensitizer and exposed breast cancer MDA-MB-231 cells to a 635 nm laser (2.86, 5.72 J/cm^2^, and 23.85 W/cm^2^). When the cells were examined 3 h after irradiation, it was discovered that this treatment induced ROS production and mediated actin fiber collapse.

Near-infrared is considered to be more non-invasive than radiation or UV, although there is a report that irradiating Chinese hamster V97-4 cells with a near-infrared laser 750 nm (25 mW) induces double strand braking of DNA (Harper et al., 2008). The intensity of this laser is not significantly different from that of lasers affecting actin polymerization in other reports. Perhaps the near-infrared light in other reports that has been shown to affect actin polymerization may also have an effect on DNA.

### Terahertz radiation

Terahertz radiation is located in the electromagnetic spectrum between infrared and microwave radiation. Yamazaki et al. reported that terahertz irradiation increased actin polymerization *in vitro* and prevented normal cell division in HeLa cells by slowing the loss of the F-actin-containing contractile ring, hence reducing normal cell division (Yamazaki et al., 2018; Yamazaki et al., 2021). However, unlike other radiation sources such as UV light, there are negative findings on the production of ROS by terahertz irradiation, indicating that processes other than ROS may be involved in the effects of terahertz irradiation on actin (Sitnikov et al., 2021).

Terahertz electromagnetic waves have been reported to cause no toxicity at all and are considered to be the most noninvasive of the methods presented in this paper. However it is reported to increase the number of micronuclei *via* an increase in actin polymerization (De Amicis et al., 2015).

### X-ray irradiation

Numerous studies have demonstrated that X-irradiation generates ROS (Ghosal et al., 2005; Kam and Banati, 2013; Srinivas et al., 2019). Additionally, multiple instances of direct actin alteration by produced ROS have been reported. After 20 min of exposure to 40 Gy of X-rays, yeast develops patches of actin, which grow into enormous actin bodies within 3 h. Since antioxidants inhibit this structure, the authors reasoned that actin is directly stimulated by the participation of radiation-induced ROS. Since a comparable actin structure generated by cysteine oxidation has been reported, this structure is believed to be induced by the oxidation of actin cysteine residues (Illner and Scherthan, 2013). In addition, it has been reported that a brief X-irradiation (5 Gy/animal) of living rats reduced actin polymerization to 33% after 3 h and then recovered after 24 h. In this study, oxidized cysteine and tryptophan residues were discovered in the actin protein. This demonstrates that ROS directly altered the polymerization of actin proteins *in vivo* (Fedorova et al., 2010a; Fedorova et al., 2010b).

X-irradiation is considered invasive to living organisms. Irradiation is known to cause genotoxicity in addition to the generation of reactive oxygen species. Radiation therapy is usually performed over a long period of time, but the papers on actin manipulation presented in this issue shows an acute actin response after a short period of irradiation, so it is not known whether genotoxicity appears afterwards.

### Chromophore-assisted light inactivation (CALI)

**FIGURE 2:**
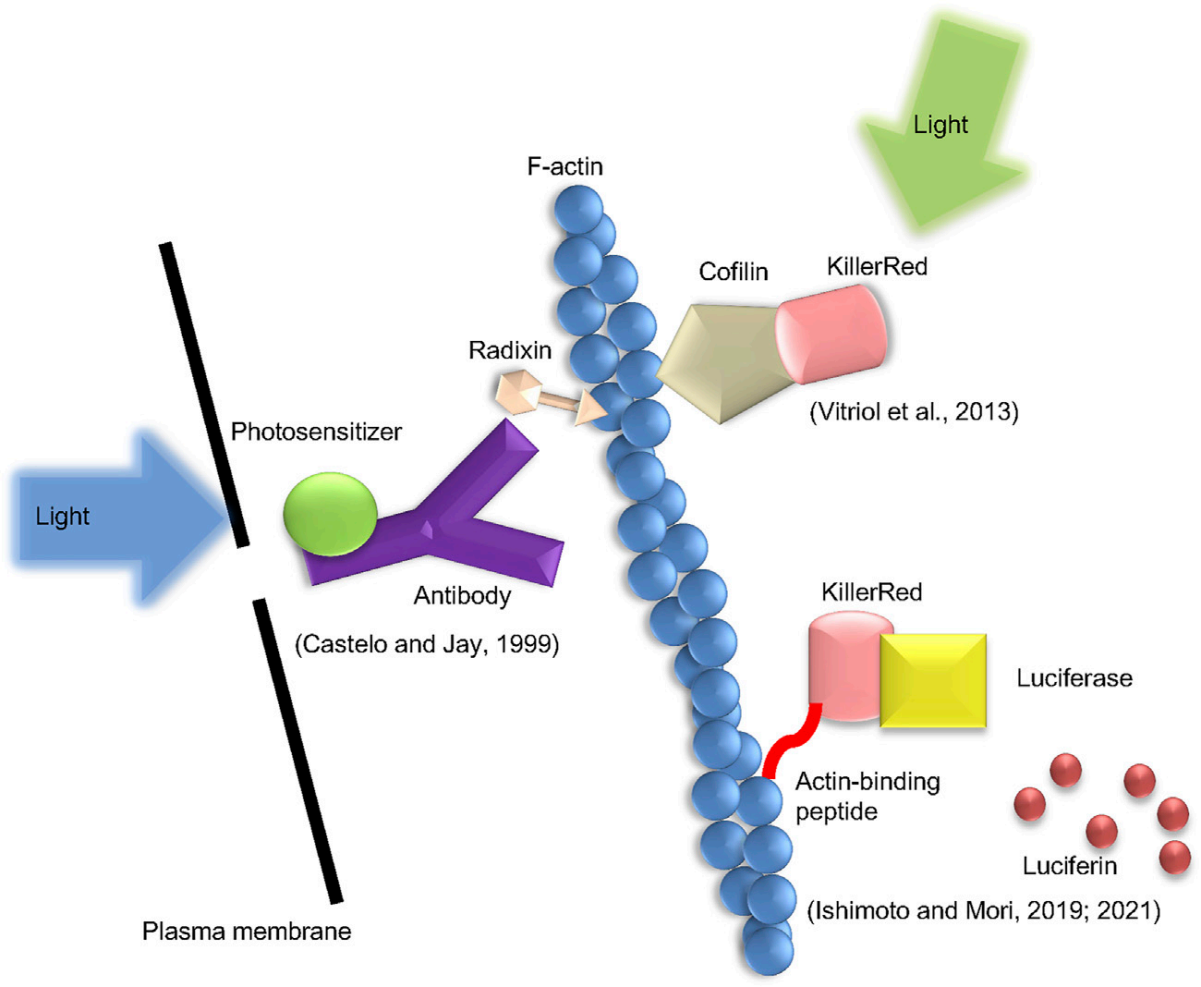
Types of the CALI method presented in this mini-review. Three types of CALI method which were introduced in the text are shown. The method by Castelo and Jay uses an anti-radixin antibody labeled with a photosensitizer and generates ROS by applying excitation light. Vitriol et al. fuse KillerRed with cofilin and express it in cells to induce ROS action on F-actin. Ishimoto et al. generate ROS using the luciferin-luciferase reaction instead of excitation light.

CALI is a method of locally generating ROS *via* photosensitizers located in the vicinity of the target (Jay, 1988), and has been applied to control actin polymerization (Figure 2). The photosensitizer is frequently employed in the antibody labeled form, and ROS are generated around the targeted protein. In principle, this approach targets plasma membrane proteins. Therefore, special techniques such as trypsin treatment are required for the antibody internalization. Radixin, a barbed end capping protein belonging to the ezrin-radixin-moesin family, was inactivated in trypsin-treated chick dorsal root ganglion neuronal growth cones by micro CALI utilizing anti-radixin antibody labeled with malachite green. This alteration decreased lamellipodia by 30% at the irradiation spot (Castelo and Jay, 1999).

In a different form of CALI, a fluorescent protein that generates ROS when exposed to excitation light is fused to a target protein and produced in the cell (Trewin et al., 2018). This permits laser irradiation to trigger the inactivation of intracellular actin-binding proteins. This group of fluorescent proteins includes miniSOG, KillerRed, and its variant, Supernova. MiniSOG is a relatively small protein composed of 106 amino acids, which emits singlet oxygen when stimulated by blue light (Shu et al., 2011). KillerRed was created by altering the hydrozoan chromoprotein anm2CP, which is known to generate superoxide in response to green light (Bulina et al., 2006a; Bulina et al., 2006b). This molecule forms a dimer, but additional amino acid modifications have produced Supernova, which does not form a dimer (Takemoto et al., 2013). Rajfur et al. reported that laser irradiation promoted stress fiber detachment from focal adhesions and subsequent stress fiber retraction in swiss3t3 cells expressing EGFP fused to actinin (Rajfur et al., 2002). Additionally, Vitriol et al. reported that CALI using cofilin-KillerRed fusion protein up-regulated actin polymerization in the lamellipodia and down-regulated the rate of retrograde flow in Cath. a-differentiated cells (Vitriol et al., 2013).

By combining firefly luciferase with the photosensitizer KillerRed, Ishimoto et al. developed a new approach for intracellular ROS production (Ishimoto and Mori, 2019, 2021). The fusion protein is expressed in cultured cells, and the luciferase-luciferin reaction is triggered by the addition of luciferin, the substrate of luciferase, followed by the excitation of KillerRed *via* bioluminescence resonance energy transfer. This approach can, so to speak, be referred to as Chemical CALI. ROS can act on F-actin by tagging this fusion protein with Lifeact, an F-actin binding protein. As a result, there was an increase in actin polymerization. It is believed that this actin structure is a cofilin-actin rod since it contains cofilin.

CALI using KillerRed and other proteins is a promising method to manipulate actin polymerization while avoiding toxicity. This is because by fusing it with the appropriate protein, it can target only specific organelles of the cell and generate ROS in a very small space. The problem with using this method intracellularly is how to express the protein in the cell and the toxicity of the expressed fusion protein itself. Adeno-associated viruses are currently candidate for noninvasive gene transfer method. In the CALI method, it has been reported that fusion of KillerRed and other proteins that migrate to the nucleus generates ROS in the nucleus and induces double strand break (House et al., 2020). This mechanism is considered to be different from the induction of double strand break by radiation or UV light.

## Discussion

Many of the papers presented here suggest that ROS produced by light or radiation exposure affect actin polymerization. However, several papers do not state that ROS generation is the cause. As a pathway other than ROS, changes in actin polymerization may be a secondary phenomenon caused by DNA damage induced by stimuli. Some reports suggest that radiation and UV induce double strand break, which enhances actin polymerization in the nucleus and cytoplasm and acts as a cellular defense mechanism (Belin et al., 2015; Osaki et al., 2016; Caridi et al., 2019; Magalhaes et al., 2020).

Compared to radiation and UV, near-infrared light is often thought to be relatively noninvasive, but it is not completely noninvasive, as reported to induce double strand breaks in DNA (Harper et al., 2008). If the purpose of manipulating intracellular actin is to induce cell death, as in radiotherapy, genotoxicity is rather welcome. However, if you want to analyze the effects of actin polymerization on cellular functions, you should make sure that the method you use does not have genotoxicity. So far, there are no reports that terahertz radiation has genotoxicity. The mechanism by which terahertz radiation affects actin is not well understood, but the fact that no DNA damage was detected suggests that it is not due to double strand break-induced polymerization. In any case, it may be an effective way to alter intracellular actin while keeping the cells alive.

Since actin and actin-binding proteins are intimately associated with cancer cell invasion and metastasis (van Helvert et al., 2018; Seetharaman and Etienne-Manneville, 2020), numerous studies suggest that they could be exploited as cancer therapeutic targets (Chen et al., 2010; Guo et al., 2013; Lee et al., 2019). However, radiation is already used in cancer therapy, and its effectiveness is based on killing cancer cells by genotoxicity. It is conceivable that more suitable applications exist for methods of controlling actin polymerization in living cells, such as those presented in this paper.

It is known that the breakdown of the structure of podocyte actin in the glomeruli of the kidney alters the structure of the glomerular capillary wall, causing proteinuria. Thus actin is considered a therapeutic target for proteinuric kidney diseases (Tian and Ishibe, 2016; Sever and Schiffer, 2018). Compounds that target actin are currently used to treat this condition (Faul et al., 2008). However, drugs that target actin may act on more than just podocytes, and there is a need for a method to manipulate actin while keeping cells alive, and the method introduced here may be effective in targeting podocytes.

There are other papers advocating therapies targeting actin and actin-binding proteins. For example, one paper suggests that targeting ROCK2, an actin-binding protein, may be therapeutic for Alzheimer’s disease by inducing autophagy, thereby reducing neurofibrillary tangles and enhancing plasticity of the spine, which is maintained in shape by actin (Weber and Herskowitz, 2021). There is also report that inducing actin depolymerization in neurons may be useful in treating methamphetamine dependence. This is based on the finding that inhibition of actin polymerization in amygdala disrupted methamphetamine-associated memories but preserved other memories. They also found that spines increased by methamphetamine-associated memories are reduced by inhibition of actin polymerization (Young et al., 2015).

The methods described in this paper are expected to be widely used not only for the treatment of diseases but also as an experimental technique in cell biology. Since the effects on actin vary depending on the cell type and stimulation parameters, and adverse reactions such as genotoxicity may occur, it is important to select the best method depending on the purpose of the experiment.
